# Editorial: Novel Insights Into the Genetics of Growth Disorders

**DOI:** 10.3389/fgene.2022.920469

**Published:** 2022-06-08

**Authors:** Mara Giordano, Liborio Stuppia

**Affiliations:** ^1^ Laboratory of Genetics, SCDU Clinical Biochemistry, University Hospital “Maggiore della Carità”, Novara and Department of Health Sciences, University of Eastern Piedmont, Novara, Italy; ^2^ Department of Psychological, Health and Territorial Sciences and Center for Advanced Sciences and Technology (CAST), G. d’Annunzio University, Chieti-Pescara, Italy

**Keywords:** growth disorder, SHOX, COL1A, Acan, Lamc3, Smad4

Although in the popular culture the definition of “genetic disease” is mainly associated to the presence of clinical signs such as intellectual disability, heart malformations or facial dysmorphisms, the history of Medical Genetics demonstrates that actually one of the first conditions associated to a genetic disease has been represented by short stature. This disorder, representing a common medical concern that paediatricians have to face in their daily practice, can be associated to different genetic conditions, with the first evidence coming from the identification of the 45, X karyotype as the cause of the Turner Syndrome (TS), characterized by short stature as one of the main clinical signs (Zinn et al., 1993). The comparison between the phenotype of TS and Klinefelter syndrome, due to a 47, XXY karyotype and characterized by tall stature, suggested the presence of one or more genes affecting human stature mapping on both X and Y sex chromosomes. Since these two chromosomes share genes only on their pseudoautosomal regions (PAR1 and PAR2), it was quite obvious to conclude that the gene(s) involved in short stature was located within these regions. Finally, in 1997, the SHOX gene, mapping within the PAR1 region, was cloned and identified as responsible both for a number of cases of idiopathic short stature and for the majority of cases of Leri-Weill dyschondrosteosis (Rao et al., 1997). In a short time, search for alterations in *SHOX* has become crucial in the molecular diagnosis of short stature (Stuppia et al., 2003; Stuppia et al., 2010; Genoni et al., 2018), in particular due to the evidence of a good response to GH treatment showed by patients with SHOX deficit (Benabbad et al., 2017). Moreover, the presence of variants affecting height also outside the *SHOX* coding region extended the spectrum of genotype–phenotype correlation in short stature (Benito- Sanz et al., 2005; Babu et al., 2021; Fanelli et al., 2022). The identification of the *SHOX* gene and its analysis in the diagnostic workflow of children with short stature prompted other researchers to investigate the role played by other genes in the pathogenesis of different forms of short stature.

Currently there are more than 200 mendelian syndromes or skeletal dysplasia associated with short stature for which the genetic cause have been identified. In some patients with short stature, variants of moderate effect explain less extreme phenotypes than those seen in disorders with classical syndromic phenotype. For example, biallelic variants in the natriuretic peptide receptor 2 (*NPR2)* (Wu et al., 2022) lead to acromesomelic displasia weather monoallelic mutations have been detected in short stature patients without any other distinctive feature (Hisado-Oliva et al., 2018) Pathogenic variants in *FGFR3*, have been found in a wide range of phenotypes, namely achondroplasia, hypochondroplasia and more recently in patients with milder forms short stature (Kant et al., 2015).

At present, a clear example of the strong interest devoted to short stature and in general to growth disorders by the international scientific community is provided by this Research Topic on *Novel Insights into the Genetics of Growth Disorders*, reporting a series of very interesting papers exploring the most relevant topics in this field.

*SHOX* and SHOX-regulated genes still represents a very promising field of study, as reported in the paper by Hoffmann et al., who analysed differentially expressed genes in SHOX-overexpressing human fibroblasts, confirming *NPPB* and *FGFR* among the most strongly regulated genes, together with 143 novel candidates. Among these, multiple Sox family members were significantly dysregulated in Shox-deficient pectoral fins highlighting an important role for these genes in Shox-related growth disorders.

Another very promising field of studies is represented by the application of the novel technologies, mainly the Next Generation Sequencing (NGS) in the identification of gene variants involved in the short stature. In this view, several papers in this Research Topic report very interesting data about the usefulness of this approach. Tang et al. investigated the genetics of 16 Chinese foetuses with skeletal dysplasia by whole exome sequencing (WES) and identified 12 cases carrying clinically relevant variants, including one deletion in *DMD* and 14 variants in other six genes. Furthermore WES allowed to detect two cases of somatic mosaicism for two distinct variants in COL1A1, one in a foetus and the other in the healthy mother of an affected carrier foetus, thus elucidating the usefulness of NGS in improving the diagnosis yield of skeletal dysplasia.

The NGS approach revealed that mutations in the aggrecan encoding gene, *ACAN*, represent one of the main causes of idiopathic and syndromic forms of short stature. Several studies retrospectively analyzed cohorts of patients that tested negative for *SHOX* alteration as the paper of Mancioppi et al., where NGS targeted analysis detected a novel *ACAN* heterozygous pathogenic variant in a family with idiopathic short stature, early-onset osteoarthritis and osteoarthritis dissecans.

The identification of novel gene variants associated with different form of growth disorders is also the topic of the two manuscripts by Li et al., Li et al. as well as of the submissions of. In the first study Li et al., authors collected clinical data and biological samples from a 12-year-old boy with Cornelia de Lange syndrome (CdLS) referred for short stature. A *de novo* pathogenic variant in *SMC3* in the proband, c.1942A>G, was evidenced by whole-exome sequencing. A review of the literature carried out by authors reported other 27 CdLS cases with variants in *SMC3*, all showing symptoms of verbal development delay and intellectual disability to different degrees. In the second paper of Li et al., the *GHR* gene was analysed in four patients with Laron Syndrome (LS). Four *GHR* variants were identified, two of which were novel mutations. Wild type and mutant GHR expression plasmids were constructed, and transiently transfected into HepG2 cells and HEK293T cells to observe the subcellular distribution of the GHR protein by immunofluorescence. It is worth underlying that as well as for other genes for which biallelic mutations cause severe syndromic forms, *GHR* is also involved in milder phenotypes such as idiopathic short stature in patients carrying monoallelic variants (Dias et al., 2017; Andrews et al., 2021).

Finally, the paper of Quian et al. investigated Occipital cortical malformation (OCCM), a disease caused by malformations of cortical development characterized by polymicrogyria and pachygyria of the occipital lobes and childhood-onset seizures, and identified novel complex heterozygous variants of *LAMC3* in a Chinese female with childhood-onset seizures. This issue also includes a study on Congenital hypertiroidism (CH), one of the most frequent endocrine disease in childhood which causes intellectual disability and short stature if untreated. Whereas most previous studies conducted on the genetic causes of CH focused on single genes, the research of Huang et al. utilized NGS to analyse a panel of 28 candidate genes related to CH. Clinically relevant variants were identified in eight genes in 14 of the 15 investigated patients (93.33%), underlying the efficiency of this approach with an important implications in clinical diagnosis and therapeutic choices.

The therapeutic perspectives in the field of short stature have also been considered in this Research Topic. The paper of Wu et al. takes into account a case of Myhre syndrome, a rare disorder caused by heterozygous mutations in *SMAD4* and characterized by dysmorphic facial features, intrauterine growth retardation, short stature, obesity, muscle hypertrophy and varying degrees of psychomotor developmental disorder. The patient, who also showed the novel symptom of giant testicles, was treated with growth hormone combined with letrozole, that successfully improved his short stature.

In conclusion, the present Research Topic provides novel information about the genetics of growth disorders, and demonstrates the usefulness of the modern technologies in disclosing novel gene variants associated to this condition.

The identification of novel genetic variants associated to growth disorders, as well as the identification of the pathogenic mechanism leading to the clinical phenotype, will improve both the diagnosis and the treatment of these disorders, leading to a “precision medicine “strategy able to identify specific solutions based on the genetic defect and avoiding a “one size fits all” approach, which can be useless, when not dangerous, for patients.
